# Comparison of Diffuse Weighted Imaging and Fluid Attenuation Inversion Recovery Sequences of MRI in Brain Multiple Sclerosis Plaques Detection

**Published:** 2017

**Authors:** Reza NAFISI-MOGHADAM, Abolghasem RAHIMDEL, Tahereh SHANBEHZADEH, Razieh FALLAH

**Affiliations:** 1 Radiologist, Department of Radiology, Shahid Sadoughi University of Medical Sciences, Yazd, Iran; 2 Neurologist, Department of Neurology, Shahid Sadoughi University of Medical Sciences, Yazd, Iran; 3 Radiologist; 4 Pediatric Neurologist, Department of Pediatrics, Growth Disorders of Children Research Center , Shahid Sadoughi University of Medical Sciences, Yazd, Iran

**Keywords:** Multiple Sclerosis, Magnetic Resonance Imaging, Diffuse Weighted Imaging, Fluid Attenuation Inversion Recovery

## Abstract

**Objective:**

Suitable magnetic resonance imaging (MRI) techniques from conventional to new devices can help physicians in diagnosis and follow up of Multiple Sclerosis (MS) patients. The aim of present research was to compare effectiveness of Fluid Attenuation Inversion Recovery (FLAIR) sequence of conventional MRI and Diffuse Weighted Imaging (DWI) sequence as a new technique in detection of brain MS plaques.

**Materials & Methods:**

In this analytic cross sectional study, sample size was assessed as 40 people to detect any significant difference between two sequences with a level of 0.05. DWI and FLAIR sequences of without contrast brain MRI of consecutive MS patients referred to MRI center of Shahid Sadoughi Hospital, Yazd, Iran from January to May 2012, were evaluated.

**Results:**

Thirty-two females and 8 males with mean age of 35.20±9.80 yr (range = 11-66 yr) were evaluated and finally 340 plaques including 127(37.2%) in T2WI, 127(37.2%) in FLAIR, 63(18.5%) in DWI and 24(7.1%) in T1WI were detected. FLAIR sequence was more efficient than DWI in detection of brain MS plaques, oval, round, amorphous plaque shapes, frontal and occipital lobes, periventricular, intracapsular, corpus callosum, centrum semiovale, subcortical, basal ganglia plaques and diameter of detected MS plaques in DWI sequence was smaller than in FLAIR.

**Conclusion:**

Old lesion can be detected by conventional MRI and new techniques might be more useful in early inflammatory phase of MS and assessment of experimental treatments.

## Introduction

Multiple Sclerosis (MS) with complexity of inflammation, demyelination, gliosis, axonal degeneration and neural loss is the most common autoimmune diseases that is typically the disease of young adults and the most common cause of nontraumatic disability in young and middle-age adults and numerous demyelination attacks in different parts of central nervous system including brain, optic nerve, spinal cord) at intervals of at least thirty days, might occur (1, 2). Different pathological evidences in MS patients lead to heterogeneity in their symptoms and clinical presentations (1). Conventional Magnetic Resonance Imaging (MRI) which includes T2-weighted, pre- and post-contrast T1-weighted scans is the basic part of McDonald criteria in diagnosis of MS. It is the most important tool in assessment of disease evolution and follow up of treatment as well (3). 

Hyper signal lesions on T2-weighted sequence of conventional MRI are characteristics of demyelinated multiple sclerosis plaques (4). However, conventional MRI cannot detect effectively damage diffusion in gray and white matters of normal appearance (1). 

Selection of suitable MRI sequences in suspected cases can help physicians to early diagnosis and decrease of MS morbidity and even mortality. It does more than sole determining whether there is a lesion in the brain and if so, how many (5). 

Approved treatments do not usually match from the view point of clinical and MRI efficacy and non-conventional magnetic resonance-derived metrics including unenhanced T1-weighted imaging, magnetized transfer imaging, proton magnetic resonance spectroscopy, diffusion-weighted imaging and functional MRI measure the destructive aspects of MS pathology selectivity, offer a better pathological substrate of the MS plaques approximation and can mark a true biology of MS seriousness (6). 

The new imaging techniques help physicians to select and determine high-risk patients in early phases of MS disorder, improve their ability to diagnose, monitor, and understand the pathophysiology of MS (7). The recent accepted brain MRI criterion for dissemination in space has a higher specificity than these lesions for multiple sclerosis versus other neurological diseases (3, 8). 

Fluid Attenuation Inversion Recovery (FLAIR) is a T2- weighted sequence, which signals that the intensity of cerebrospinal fluid is suppressed and the conspicuity of periventricular lesions is increased. Disadvantages of FLAIR sequences are less sensitivity in showing of brainstem and cerebellum plaques, cerebrospinal fluid flow artifacts and long needed time for imaging (4). 

To evaluate neurological disorders, diffuse weighted imaging (DWI) is used which is a reference quantitative MRI technique that can detect specifically important MS white matter pathology. Diffusion measures water molecules motion in brain white matter, which can be halted by cellular structures, include myelin fiber tracts, cell membranes and axonal cytoskeletons (1, 9). 

Active perivascular inflammatory lesions can be shown in MRI with contrast and DWI sequence of MRI can distinguish sensitively acute ischemic cerebral events and diffusion alterations in active inflammatory lesions and when usage of contrast drugs is contraindicated , DWI might be essential for timely and correct multiple sclerosis diagnosis (10). 

The purpose of this study was to compare effectiveness of diffuse weighted imaging and FLAIR sequences in detection of brain multiple sclerosis plaques. 

## Material & Methods

In this analytic cross sectional study, DWI and FLAIR sequences of without contrast brain MRI of consecutive patients with final diagnosis of MS referred to MRI Center of Shahid Sadoughi Hospital, Yazd, Iran from January to May 2012, were evaluated. 

Sample size was assessed as 40 People based on Z formula and a confidence interval of 95% with 80% power, S= 30 and d=15 to detect any significant difference between the two sequences with a level of 0.05. 

The patients were recruited from outpatient MS Clinic of Shahid Sadoughi University of Medical Sciences, Yazd, Iran. Eligible participants included those who had MS based on the 2005 revised International Panel (McDonald) criteria (3) in clinical evaluation by the neurologist of research after two or more clinical attacks of CNS demyelinating events and objective clinical evidence of two or more lesions as defined by the McDonald Criteria and exclusion of other possible alternative diseases (such as acute disseminated encephalomyelitis or neuromyelitis optica) (10). 

Informed consent was taken from patients before taken of MRI and the study has been approved by the Ethics Committee of Shahid Sadoughi University of Medical Sciences, Yazd, Iran. 

Patients with medical history of other neurologic or vascular disorders, who had standard contraindications for MRI (claustrophobia and presence of any metal prosthesis in their body because of previous medical interventions) and those with Parkinsonism (due to impairing in image quality) were excluded. 

MRI machine characteristic in the hospital was the Magnetom Avanto 1.5T magnetic resonance scanner and manufactured by Siemens in 2011. 

T1 weighted imaging (WI) and T2WI, FLAIR and DWI sequences of MRI of the patients were interpreted by a radiologist of research. Apparent diffusion coefficient (ADC) maps were derived automatically from diffusionweighted MR imaging. 

In radiological assessment, total number of brain MS plaques , biggest plaque size, plaque shape (oval, circular, linear and amorphous) and plaque location [ lobar white matter (frontal , parietal temporal and occipital), periventricular white matter, internal capsule, corpus callosum, centrum semioval, subcortical, cerebellum, basal ganglia, thalamus and brain stem] were assessed. Variables such age, sex, clinical symptoms and MRI characteristics, were reviewed. The data were analyzed using SPSS version 17 (Chicago, IL, USA). Chi-square test was used for data analysis of qualitative variables and mean values were compared using independent t-test. Differences were considered significant at P values of less than 0.05. The sensitivity and specificity of the DWI for prediction of the demyelinating lesions on FLAIR measured. 

## Results

Thirty-two females (8%) and 8 males (20%) with mean age of 35.20 ± 9.80 yr (range = 11-66 yr) were evaluated. Table1 shows the duration of disease and frequency of clinical symptoms based on gender which indicates the duration of the disease was not statistically significantly different in both genders and blurred vision and numbness were the most frequent symptom in females and males, respectively. Vertigo was statistically significantly more frequent in females (P = 0.03) . 

The 30 without contrast brain MRI evaluated and totally, 340 demyelinating lesions and MS plaques including 127(37.2%) in T2WI, 127(37.2%) in FLAIR, 63(18.5%) in DWI and 24(7.1%) in T1WI were detected.

Oval [N=104(30.5%)] and round [N= 100(29.3%)] shapes were the most frequent shapes of brain plaque and shape of 63(18.5%), 47(13.8%) and 27(7.9%) of brain plaques were amorphous, continues and linear, respectively.

Frequency of different shapes of brain plaque based on different MRI sequences is present in Table 2 that shows FLAIR and T2WI sequences are more efficient than DWI in detecting of brain MS plaques and also in detecting of oval, round and amorphous plaque shapes. The most frequent plaque shape in T2WI sequence is round and in other MRI sequences is oval. 

The sensitivity, specificity, positive predictive value and negative predictive value of the DWI for prediction of the demyelinating lesions on FLAIR were 85.7%, 76.4%, 41.2% and 84%, respectively.

 The mean ADC in the oval lesion was 2.32± 0.16 × 10(- 3) mm (2)/s and round lesions had a mean ADC of 2.11 ± 0.11 × 10(-3) mm (2)/s. 

Location and diameter of brain MS plaques in different MRI sequences is shown in Table 3 that indicates FLAIR sequence was more efficient than DWI in detecting of frontal and occipital lobes, periventricular, intracapsular, corpus callosum, centrum semiovale, subcortical and basal ganglia MS plaques and diameter of detected brain MS plaques in DWI sequence was smaller than FLAIR and T2WI. 

**Fig 1:**
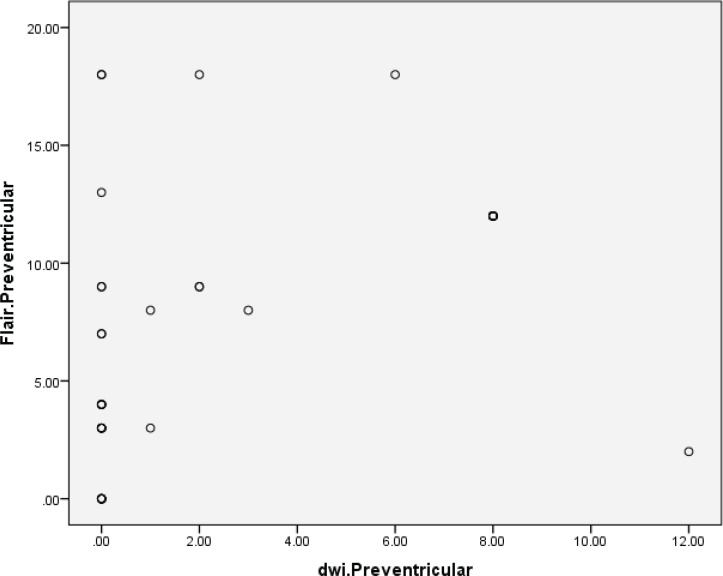
Comparison of FLAIR and DWI sequences in depiction of periventricular plaques

**Fig 2:**
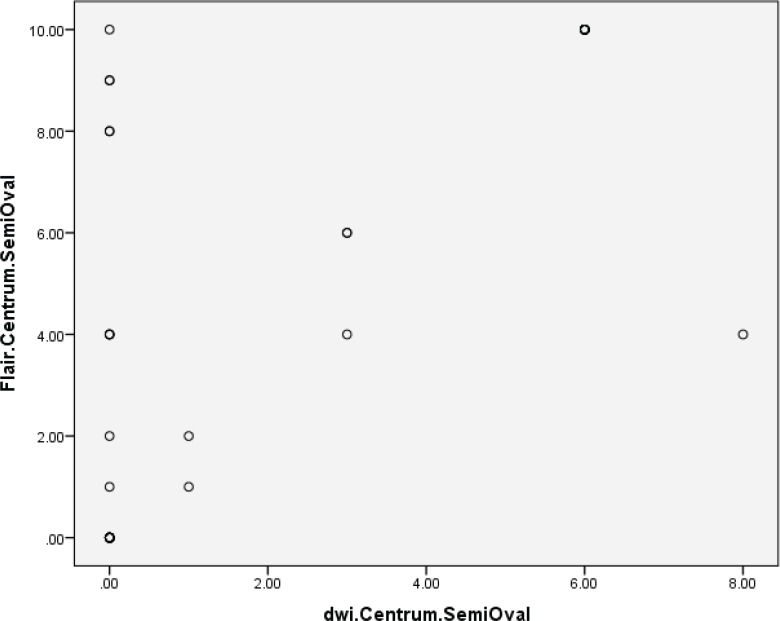
Comparison of FLAIR and DWI sequences in depiction of centrum semiovale plaques

Figure 1 and 2 compare FLAIR and DWI sequences in depiction of periventricular and centrum semiovale plaques, indicative that there was no correlation between the two sequences of MRI.

**Table 1:** Comparison of Duration of Disease and Frequency of Clinical Symptoms Based on Gender

**Data**		**Female** **N(%)**	**Male** **N(%)**	**P.** **value**
**Sex**				
**Duration of disease**	less than one year	6(18.7%)	1(12.5%)	0.28
	1-5 years	8(25%)	5(62.5%)	
	5-10 years	8(25%)	1(12.5%)	
	10-14 years	10(31.3%)	1(12.5%)	
**C** **linical symptoms**	Ataxia	6(18.75%)	0	0.18
	Ptosis	1(3%)	0	0.61
	Diplopia	8(25%)	3(37.5%)	0.48
	Vertigo	12(37.5%)	0	0.03
	Numbness	20(62.5%)	5(62.5%)	1
	Headache	17(53%)	2(25%)	0.15
	Muscle weakness	14(43.75%)	4(50%)	0.75
	Blurred vision	24(75%)	4(50%)	0.17
	Muscle spasm	13(40.7%)	1(12.5%)	0.14
**Total number of patients**		32	8	

**Table 2:** Frequency of Different Shapes of MS Plaque Based on MRI Sequences

**MRI sequences**	**T1WI** **N(%)**	**T2WI** **N(%)**	**FLAIR** **N(%)**	**DWI** **N(%)**	**Total**	**P.** **value**
**Shape**						
**Oval**	11(10.6%)	35(33.6%)	39(37.5%)	19(18.3%)	104(100%)	0.01
**Round**	11(11%)	36(36%)	35(35%)	18(18%)	100(100%)	0.01
**Linear**	0	14(51.8%)	13(48.2%)	0	27(100%)	0.89
**Amorphous**	2(3.2%)	25(39.7%)	24(38.1%)	12(19%)	63(100%)	0.03
**Confluent**	0	17(36.1%)	16(34.1%)	14(29.8%)	47(100%)	0.1
**Total**	24(7.1%)	127(37.2%)	127(37.2%)	63(18.5%)	341(100%)	0.02

**Table 3:** Location and Diameter of Brain MS Plaques in Different MRI Sequences

**MRI sequences**		**T1WI** **N(%)**	**T2WI** **N(%)**	**FLAIR** **N(%)**	**DWI** **N(%)**	**Total** **N(%)**	**P.** **value**
**Location**							
**Lobe**	**Frontal**	1(4.2%)	2(1.6%)	2(1.6%)	2(3.2%)	7(2 %)	0.001
	**Parietal**	0	1(0.8%)	2(1.6%)	2(3.2%)	5(1.5%)	0.2
	**Temporal**	0	2(1.5%)	2(1.6%)	2(3.2%)	6(1.7%)	0.1
	**Occipital**	0	2(1.5%)	1(0.8%)	1 (1.68%)	4 (1.2%)	0.01
**Periventricular**		10 (41.6%)	26(20.5%)	28(22 %)	16(25.4%)	80 (23.5%)	0.001
**Intracapsular**		1(4.2%)	4(3.2%)	6(4.7%)	0	11(3. 2%)	0.01
**Corpus callosum**		1(4.2%)	20(15.7%)	18(14.2%)	12(19%)	51(15%)	0.03
**Centrum semiovale**		6 (25%)	20(15.7%)	18(14.2%)	14(22.2%)	58 (17.7%)	0.02
**Subcortical**		5(20.8%)	14 (11%)	18(14.2%)	4(6.3%)	41 (12%)	0.001
**Basal Ganglia**		0	12(9.5%)	16 (12.6%)	5(8%)	33(9.7%)	0.01
**Thalamus**		0	12 (9.5%)	4 (3.2%)	0	16 (4.7%)	0.3
**Brainstem**		0	12(9.5%)	12 (9.5%)	5(83%)	29 (8.5%)	0.4
**Plaque diameter in millimeter (mean ± SD)**		11.43±4.6	11.24±5.74	11.78±5.48	9.87±5.06	-	0.001

## Discussion

In the present study, DWI and FLAIR sequences of brain MRI of MS patients were reviewed and the results showed that FLAIR sequence was more efficient than DWI in detecting of brain MS lesions, ovoid plaques and lesions in periventricular area, centrum semiovale and corpus callosum that is in compliance with Miabi et al. study in Tabriz, Iran (11). In this study, diameter of brain MS plaques in DWI sequence was smaller than FLAIR. However, in another Iranian study, minimum plaque size was significantly lower in FLAIR sequence (12). 

Conventional MRI sequences consist of proton density; T1WI, T2WI and FLAIR are valuable tools to assess MS activity over time and are now routinely used and accepted in diagnosis and detection of therapeutic effects and extension of clinical observations in MS patients (4, 13, 14). However, different pathologies such inflammation, Wallerian degeneration, demyelination, edema, and axonal loss cannot be recognized by T2WI and T1WI sequences of conventional MRI. Advanced metrics MRI techniques cause a better understanding of the most likely related to the disease activity pathologic processes as well as their clinical progression and is able to show a range of tissue changes such as demyelination, axonal loss, deposition of iron, and neurodegeneration. Results of present study showed that FLAIR sequence of conventional MRI detected more brain MS plaques than DWI sequence as a new technique. The explanation for this discrepancy is difference in specificity of these techniques. Besides DWI is more specific in detection of acute and active brain lesions in early inflammatory phase of MS, old lesion and irreversible tissue damage within and outside MS lesions can be detected better by sequences of conventional MRI (4, 12, 14). Due to lack of standardization, the new techniques have not been used in the MRI consensus guidelines for brain and spinal cord imaging in MS patients (13). DWI is a quantitative MRI technique that can detect tissue injury outside T2-visible lesions, i.e., in the so-called normal appearing white and gray matters of MS patients at different stages of the disease and depicting of acute cerebral ischemia. It is useful while ischemic cerebral events is in the differential diagnosis and DWI may detect lesions that are not obvious by routine methods (11, 12, 15, 16). 

DWI was one of important diagnostic and effective modalities in patients with MS and findings of DWI sequence are correlated with disease severity, duration and disability (17). 

In present study, the most frequent plaque shape in T2WI sequence was round and in other MRI sequences was ovoid. In a Chinese study, also, most of the MS plaques were round or oval (18). 

Most of plaques, showed in this study, were located in periventricular, centrum semiovale and corpus callosum, in consistent with the finding of other studies (11, 12, 18). 

Limitation of the study was not returning of some patients and missing of their MRI data.

**In conclusion**, FLAIR sequence of conventional MRI can detect more brain MS plaques than DWI sequence as a new technique. Old lesion and irreversible tissue damage can be detected by conventional MRI and new MRI techniques might be more useful in early inflammatory phase of multiple sclerosis and assessment of experimental treatments. Other next studies with more sample size of MS patients, evaluation and comparison of different MR techniques and in different stages of the disease might be useful in selection of better and suitable MRI protocols in detecting and diagnosis of MS plaques and screening of high risk patients. 
